# Tubulonodular Pericallosal Lipoma With Callosal Dysgenesis in an Adult Presenting With a Lapse in Consciousness: A Case Report

**DOI:** 10.7759/cureus.96431

**Published:** 2025-11-09

**Authors:** Ayomide O Ayo-Salami, Godknows Osarhiaekhimen, Adeolu Morawo

**Affiliations:** 1 Neurology/Internal Medicine, American University of Integrative Sciences, Atlanta, USA; 2 Internal Medicine, Tertiary Education Trust Fund, Abuja, NGA; 3 Neurology/Vascular Neurology, Creighton University School of Medicine, Grand Island, USA

**Keywords:** corpus callosum, dysgenesis, intracranial lipomas, pericallosal lipoma, seizure

## Abstract

Intracranial lipomas are rare congenital anomalies that result from the abnormal development of meningeal tissues, causing the formation of fatty deposits within the intracranial cavity. The pericallosal region is the most frequent site where such lipomas are often associated with structural abnormalities of the corpus callosum. Although many cases remain clinically silent, symptomatic presentations can include seizures, headaches, cognitive impairment, or unusual neurobehavioral findings. We present the case of a 30-year-old woman who was evaluated in the emergency department after a motor vehicle accident, which was preceded by a transient loss of consciousness, presumed to be a seizure. Neuroimaging revealed a tubulonodular pericallosal lipoma associated with callosal dysgenesis.

## Introduction

Intracranial lipomas, arising from aberrant differentiation of primitive meningeal mesenchyme, constitute an exceedingly rare category of brain tumors, comprising less than 1% of all intracranial neoplasms. These lipomas are most frequently located in the corpus callosum and occur equally among males and females [1]. Given their composition of ectopic adipose tissue, it is terminologically more accurate to designate these anomalies as choristomas rather than true neoplasms [1]. These lesions are intimately associated with the inner stratum of the pia-arachnoid-dural complex [2,3]. They exhibit a predilection for the midline, particularly adjacent to the pericallosal cistern. Less frequent locations include the quadrigeminal plate, superior cerebellar peduncle, suprasellar cistern, cerebellopontine angle cistern, and sylvian cistern [2,4]. Sylvian fissure lipomas are a pertinent consideration in the differential diagnosis of epileptic patients. Midline anomalies and concomitant malformations, such as aneurysms, are often coexistent with symptomatic intracranial lipomas. Seizures are the most common presenting symptom of corpus callosum lipomas, although they are directly attributable to the lipoma in only a minority of cases [5]. Hence, the significance of these lipomas remains unclear. This case report aims to establish a clinical correlation between the presence of a tubulonodular pericallosal lipoma and the occurrence of a seizure in an adult, highlighting the potential pathogenic relationship between this rare intracranial lesion and seizure activity.

## Case presentation

A 30-year-old previously healthy obese Hispanic woman was brought to the emergency department by emergency medical services following a rear-end motor vehicle collision, during which she was the restrained driver. Airbags were deployed, there was no windshield damage, and no prolonged extrication was required. Upon arrival, she was nonverbal and disoriented, raising concerns for a possible neurological event preceding the accident.

On initial examination, she appeared confused but had stable vital signs (temperature 36.4°C, blood pressure 114/58 mmHg, heart rate 79 bpm, respiratory rate 19/min, and oxygen saturation 97% on room air). A cervical collar was in place. Cardiopulmonary and abdominal examinations were normal, and there was no evidence of acute trauma or airway compromise.

**Figure 1:**
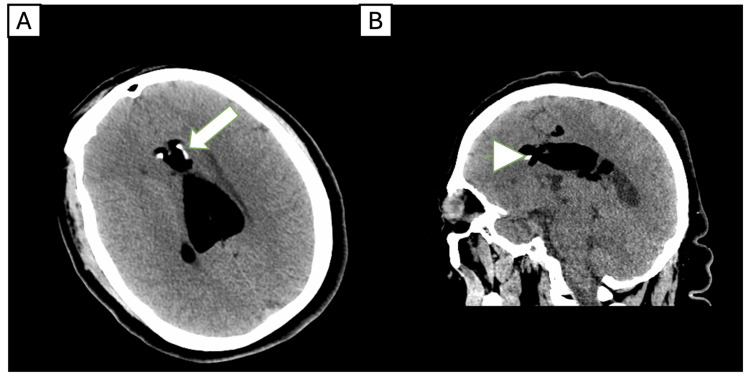
Non-contrast head CT was performed in 2024. Axial (A) and sagittal (B) images reveal a large, well-defined lipomatous lesion along the corpus callosum, demonstrating peripheral calcifications. The findings are consistent with a calcified pericallosal lipoma. CT: computed tomography

**Figure 2:**
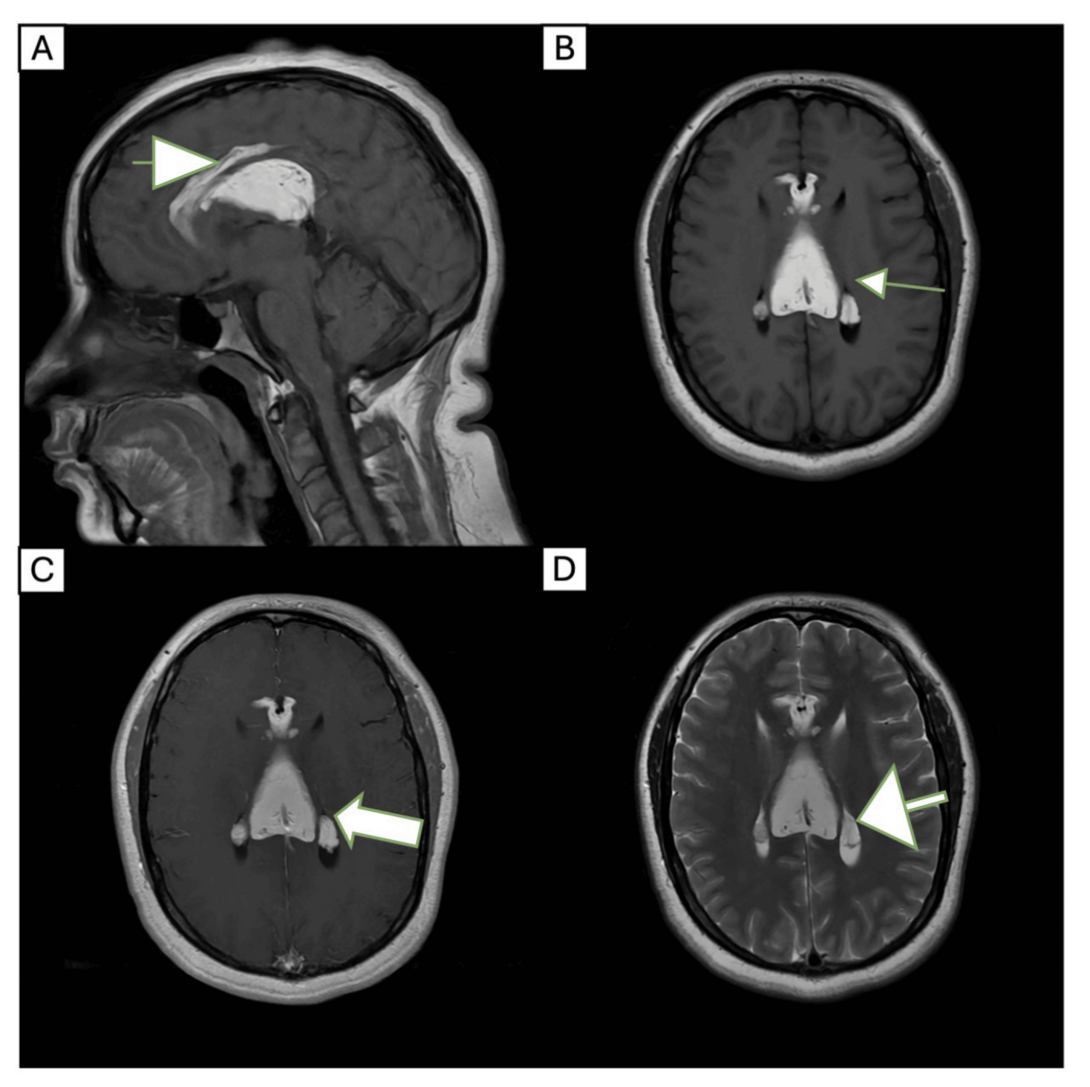
MRI brain with and without contrast was performed in 2024. Sagittal T1-weighted image (A), axial T1-weighted image (B), axial T1 post-contrast (C), and axial T2-weighted image (D) demonstrate a well-circumscribed, non-enhancing midline fatty mass measuring 6.1 × 3.3 cm. The lesion is located along the corpus callosum and is associated with callosal dysgenesis, consistent with a diagnosis of pericallosal lipoma. MRI: magnetic resonance imaging

A non-contrast cranial computed tomography (CT) scan revealed a peripherally calcified fatty mass along the corpus callosum, suspicious for a lipoma (Figure 1). Further evaluation with magnetic resonance imaging (MRI) with and without contrast demonstrated a well-circumscribed tubulonodular pericallosal lipoma measuring 6.1 × 3.3 cm, associated with callosal dysgenesis (Figure 2). The lesion showed no enhancement and no significant mass effect. An electroencephalogram (EEG) performed during hospitalization yielded normal findings.

Additional trauma imaging (CT cervical spine, chest, abdomen, and pelvis) revealed no acute fractures or visceral injury. Laboratory studies were notable for mild hypokalemia (3.2 mmol/L), which was corrected with electrolyte supplementation. Other values were unremarkable (Table 1). Urine drug screen and blood alcohol were negative, and there was no evidence of toxic ingestion. She was started on analgesics, a muscle relaxant, and deep venous thrombosis prophylaxis with heparin during admission.

**Table 1:** Laboratory parameters * outside the reference (normal) range WBC: white blood cells

Parameter	Day 1	Day 2	Reference range
Sodium (mmol/L)	136	138	135–145
Potassium (mmol/L)	3.2*	3.7	3.5–5.0
Creatinine (mg/dL)	0.48*	0.52	0.7–1.2
Calcium (mg/dL)	8.7	8.1*	8.6–10.2
WBC (k/µL)	7.7	7.9	4.0–10.0
Hemoglobin (g/dL)	13.2	12.7	12–16

By the following morning, the patient had regained full alertness and was able to communicate through a Spanish-language interpreter. She reported no memory of the accident and denied prior seizures, blank stares, tongue biting, or involuntary movements. She was not being managed for any chronic medical condition or on any long-term medication. She reported no family history of presenting symptoms or neurological disorders. The systematic review was not contributory. Vital signs were stable, and neurological examination was normal.

Given the imaging findings, a neurosurgical consultation was obtained. Surgical intervention was deemed unnecessary, and conservative management was recommended. Antiseizure pharmacotherapy was advised, but the patient declined treatment. She was discharged the following day in stable condition with outpatient neurology and neurosurgery follow-up.

At her one-month neurosurgical follow-up, she reported occasional frontal headaches but no prodromal symptoms of seizures or further neurological symptoms. Clinical examination remained normal. A repeat MRI confirmed a classic midline pericallosal lipoma without hydrocephalus. She was counseled on conservative management, with plans for repeat imaging in six months and instructions to return for any worsening symptoms. At the neurology clinic follow-up six months post-discharge, the patient remained stable, with no interval seizures.

## Discussion

Intracranial lipomas are rare congenital malformations believed to result from the incomplete resorption of the primitive meninx between the 8th and 10th weeks of gestation, leading to aberrant differentiation into adipose tissue [6]. These lesions are typically located in the interhemispheric fissure and are frequently associated with developmental anomalies of the corpus callosum [7]. Histologically and radiologically distinct from adjacent neural structures, these lesions support a classification as malformations (choristomas) rather than true neoplasms [8].

Pericallosal lipomas are subclassified based on morphology and location. The tubulonodular type, as observed in this case, typically presents anteriorly, measures more than 2 cm, and is more frequently associated with callosal dysgenesis. In contrast, the curvilinear variant is usually smaller, located posteriorly, and exhibits fewer associated anomalies [1,2].

Although often discovered incidentally, pericallosal lipomas can present with a range of neurological symptoms, including new-onset seizures, headaches, and neuropsychiatric disturbances. The pathophysiological basis for such symptoms remains poorly defined, and seizure activity may not be directly attributable to the lipoma itself. In fact, only a subset of cases demonstrates a clear epileptogenic focus on the EEG [9]. In our patient, EEG findings were unremarkable, and no prior seizure history was elicited. Nonetheless, the clinical context, a transient lapse in consciousness followed by a traumatic event, raises the possibility of a previously unrecognized seizure. Other differentials of a transient lapse in consciousness include a cerebrovascular accident, hypoglycemia, arrhythmia, traumatic brain injury, and alcohol intoxication, all of which were ruled out by patient history, clinical examination, imaging, and blood work.

Although the causality between pericallosal lipomas and seizure activity remains uncertain, this case contributes to the emerging literature suggesting that these stable congenital anomalies may present symptomatically in adulthood. In this case, other differentials for seizures include electrolyte abnormalities, mass lesions in the brain, substance intoxication, and withdrawal, which were inconsistent with our findings.

Radiographically, pericallosal lipomas are typically well-demarcated, non-enhancing, and hyperintense on T1-weighted MRI sequences. Signal attenuation on fat-suppressed sequences confirms their adipose nature, and no associated mass effect or edema is typically observed. In our case, MRI findings were consistent with a benign fatty lesion [10,11].

Management of pericallosal lipomas is typically conservative. Treatment is reserved for symptomatic cases, with seizure control achieved through standard anticonvulsant therapy. Surgical resection is generally discouraged due to high perioperative risk and limited benefit, particularly in the absence of mass effect or intractable symptoms [12]. In this instance, the patient declined anticonvulsant therapy and was referred for neurosurgical evaluation, with imaging follow-up planned in six months.

## Conclusions

Pericallosal lipomas represent a rare subset of intracranial lipomas, often discovered incidentally, and are associated with congenital malformations, such as callosal dysgenesis. While typically asymptomatic, they may present with seizures or other neurologic disturbances in adulthood, particularly in the context of stressors such as trauma. This case highlights the importance of considering pericallosal lipomas in the differential diagnosis of first-time seizures in adults, even when the lesion is longstanding and presumed benign.

MRI remains the diagnostic modality of choice, allowing for accurate characterization of lesion morphology and associated anomalies. Differential diagnoses include fatty falx cerebri, dural venous sinus thrombosis, and angiomyolipomas. Management is usually non-operative, with symptomatic treatment tailored to individual clinical presentations. Surgical intervention is rarely indicated and should be reserved for cases with refractory symptoms or significant mass effect. Continued reporting of such cases is essential to define better the clinical relevance and optimal management of these rare entities.
